# Peru History of Coca

**Published:** 1902-05

**Authors:** 


Peru History of Coca. "The Divine Plant" of the Incas. With an introductory account of the Incas, and of the Andean Indians of today. By W. Golden Mortimer, M. D., Fellow of the New York Academy of Medicine; Member of the Medical Society of the County of New York; Member of the New York Academy of Sciences; Member of the American Museum of Natural History; formerly Assistant Surgeon to the New York Throat and Nose Hospital, etc. Octavo, pp. xxxii.—576. With 178 illustrations. New York: J. H. Vail & Co. 1901. [Price, $5.00 net.]

This history—it should be called a story—is a most interesting piece of work and bears all the evidences of painstaking and searching preparation. Dr. Mortimer has added to the literature of the world a historical work which will repay careful study. The title is and is not misleading, for while the book tells all that is known of the divine plant of the Incas from earliest time and its uses by the Indians, it also gives a most interesting account of the Andean Indians of today. It is the most extensive and exhaustive work on the subject which ever has been written. Interest is added to the purely therapeutic side of the book by tales of travel and adventure and conquest which were necessary to give to science the use of coca.

The origin and uses of the divine plant are traced from the earliest accounts which are to be found. The story begins with the Incas and follows along the life of these remarkable people until their overthrow by the Spanish. It tells of their industries, religious rites, science, arts and potteries. Full of life and color it commands admiration. Dr. Mortimer spent years in the preparation of the book and is deserving of the highest praise for the interesting story he has given us. The book is profusely illustrated. N. W. W.

